# Discordance in diagnostic assessment of Achilles tendon thickening between soft X-ray radiography and ultrasonography among patients with coronary artery disease

**DOI:** 10.1038/s41598-026-49444-9

**Published:** 2026-04-21

**Authors:** Tadashi Itagaki, Yasushi Ueki, Yushi Oyama, Junko Iguchi, Koki Fujimori, Daisuke Sunohara, Yuki Yamamoto, Yoshiteru Okina, Hidetomo Nomi, Tamon Kato, Tatsuya Saigusa, Kyuhachi Otagiri, Soichiro Ebisawa, Koichiro Kuwahara

**Affiliations:** 1 Department of Cardiovascular Medicine, Shinshu University School of Medicine, Matsumoto, Japan; 2 Department of Cardiology, Ina Central Hospital, Ina, Japan

**Keywords:** Achilles tendon thickness, Soft X-ray radiography, Ultrasonography, Familial hyperlipoproteinemia, Coronary artery disease, Cardiology, Diseases, Medical research

## Abstract

Achilles tendon (AT) xanthomas are characteristic manifestations of familial hypercholesterolemia (FH). Although soft X-ray radiography (XR) and ultrasonography (US) are used to assess AT thickness, data comparing their diagnostic concordance are limited. We evaluated the correlation and diagnostic agreement between XR and US in measuring AT thickness in coronary artery disease (CAD) patients. We prospectively enrolled CAD patients undergoing AT thickness measurement by XR and US between April 2024 and March 2025. AT thickening was defined according to the 2022 Japan Atherosclerosis Society guideline (XR: ≥8/7.5 mm [male/female], US: ≥ 6/5.5 mm [male/female]). Among 262 patients (14 [5.3%] clinically diagnosed FH), median AT thickness by XR was greater than that by US (7.0 [IQR 6.3–7.6] mm vs. 5.8 [IQR 5.1–6.1] mm, *P* < 0.001). XR and US measurements were significantly correlated (*r* = 0.632, *P* < 0.001). The concordance rate for AT thickening was 83.2% and the discordance (16.8%) was primarily due to XR–/US+ cases (14.9%). In conclusion, although there was a significant correlation between XR and US for AT thickness measurement, discordance in the diagnosis of AT thickening was observed in 17% of patients. Physicians should be aware that US assessment may result in more patients diagnosed with AT thickening than XR.

**Supplementary Information:**

The online version contains supplementary material available at 10.1038/s41598-026-49444-9.

## Introduction

Familial hypercholesterolemia (FH) is a common yet frequently underdiagnosed autosomal dominant disorder, affecting 1 in 30 individuals with coronary artery disease (CAD) globally^1^. FH is characterized by a lifelong elevation of low-density lipoprotein cholesterol (LDL-C) and the development of tendon xanthomas^2^. If left untreated, FH leads to early-onset atherosclerosis and substantially increases the risk of cardiovascular events, highlighting the importance of early diagnosis and treatment^2^.

Extensor tendon xanthomas (Achilles, subpatellar, and hand extensor tendons) have histological features that resemble foam cell formation and lipid accumulation in atherosclerotic plaques^3^. Several FH diagnostic criteria include tendon xanthomas as physical findings specific for FH^4–6^. Achilles tendon (AT) thickening is widely recognized as an early characteristic of xanthomas and one of the diagnostic criteria for FH in the Japanese Atherosclerotic Society (JAS) FH guideline^6^.

Soft X-ray radiography (XR) and ultrasonography (US) are used for the assessment of AT thickening. Although XR has been widely used for AT thickness assessment, its limited image resolution results in high measurement variability. In contrast, US has better imaging quality and provides more accurate AT thickness measurements. A previous study determined the cut-off value for AT thickening using US to discriminate genetically diagnosed FH^7^; however, data directly comparing the diagnostic agreement between XR and US for AT thickening, based on the cut-off values endorsed by the JAS FH 2022 guideline, remain lacking.

Therefore, we aimed to assess the correlation and diagnostic agreement between XR and US for AT thickness measurement in patients with CAD.

## Methods

### Study population

Consecutive CAD patients undergoing AT thickness assessment were enrolled into the Achilles Study, a prospective, multicenter, observational study (UMIN 000053786)^8^. For the current study, patients with CAD undergoing both XR and US for AT measurements between April 2024 and March 2025 were analyzed. Patients not available for AT thickness measurements by XR or US were excluded. The diagnosis of FH was based on the criteria of the JAS FH 2022 guideline^6^. FH was clinically diagnosed if two or more of the following criteria were fulfilled: (1) Elevated LDL-C levels (untreated LDL-C levels ≥ 180 mg/dL), (2) Presence of tendon xanthomas (dorsal hand, elbow, knee, etc. or Achilles tendon thickening) or cutaneous nodular xanthomas, (3) Family history of FH or premature CAD (first-degree relatives)^6^. Genetic testing for FH was performed at physician’s discretion. This study was performed in accordance with the principles of the Declaration of Helsinki. Given the observational design of the study, the requirement for written informed consent was waived.

### AT thickness measurement

XR for the AT thickness assessment was performed according to the JAS guideline^6^. Briefly, XR were obtained with each ankle positioned lateral side down on the film and the foot flexed at 90°, and the anteroposterior diameter of both AT was measured at the point of greatest thickening, excluding subcutaneous tissue. AT thickness measurements were performed by the cardiologists (I.T. and O.Y.) who were blinded to the clinical data of the patients and AT thickness measurements by US. US was performed according to the recommendations of JAS guideline^6^. In brief, participants were positioned on the bed in either a kneeling or supine posture. The probe was positioned perpendicular to the midline of the foot, with the angle between the probe and the skin maintained at approximately 90°. Tendon thickness was measured along the direction of maximal thickening rather than in the anteroposterior direction on the short-axis image. The long-axis image was assessed in the same manner, accounting for tendon torsion and ensuring that the region of maximal thickening was visualized for measurement. AT thickness was measured at the point of greatest thickening. AT structural abnormalities were defined as the presence of calcification, abnormal layer structure, and localized hypoechogenicity. The testing equipment and US probes included an Aplio i700 system (Canon Medical, Otawara, Japan) with a high-frequency linear probe operating at a central frequency of 18 MHz, as well as a LOGIQ E10 system (GE Healthcare Japan, Tokyo, Japan) with a high-frequency linear probe operating at a central frequency of 15 MHz. The measurements were performed by the sonographers who were blinded to the clinical data of the patients and AT thickness measurements by XR. The greater value of AT thickness between the right and left sides by each modality was used for patient-level analysis. The measurements of each AT by XR and US were compared for AT-level analysis. ATs with a history of tendinitis or previous trauma were excluded from measurement. The cut-off values for AT thickening were defined according to the JAS FH 2022 guideline (XR: ≥ 8 mm in male and ≥ 7.5 mm in female, US: ≥ 6 mm in male and ≥ 5.5 mm in female)^6^.

### Statistical analysis

Continuous variables were summarized as median (interquartile range [IQR]) due to the non-normal distribution of the data and were compared using the Mann–Whitney U test, whereas categorical variables were expressed as numbers and percentages and compared using Fisher’s exact test. AT thickness by XR and US was compared using the Wilcoxon signed-rank test, and AT thickening detection rate was assessed using McNemar’s test between the two modalities. Spearman’s rank correlation coefficient was calculated to investigate the correlation between AT thickness by XR and US. Agreement between XR and US measurements of AT thickness was evaluated using the Bland–Altman analysis, with mean differences and 95% limits of agreement calculated. In addition, Cohen’s kappa coefficient (κ) was used to evaluate the agreement between the two modalities for the diagnosis of AT thickening. Receiver operating characteristic (ROC) curve analysis was performed to evaluate the diagnostic performance of US for AT thickening by XR as the reference, and the areas under the curve (AUCs) were calculated. Logistic regression analysis was conducted to investigate the factors associated with the discordance between XR and US for AT thickening. The inter-rater reliability of AT thickness measurements was assessed in 40 randomly selected XR images and 11 randomly-selected patients (5 at Shinshu University Hospital and 6 at Ina Central Hospital) undergoing US using the intraclass correlation coefficient (ICC). In addition, the inter-rater reliability for the assessment of AT structural abnormalities was evaluated in 40 randomly selected patients (20 at Shinshu University Hospital and 20 at Ina Central Hospital) using the kappa coefficient. *P* values were two-tailed and considered under 0.05 as statistically significant. All statistical analyses were performed using R (The R Foundation for Statistical Computing, Vienna, Austria) and EZR (Saitama Medical Center, Jichi Medical University, Saitama, Japan)^9^.

## Results

### Study population

The patient characteristics are summarized in Table 1. Among 262 patients, the median age was 75 years and 84.4% of patients were male. Dyslipidemia was observed in 61.1%, family history of premature CAD in 7.7%, and clinically-diagnosed FH in 5.3%. The distribution of fulfilled diagnostic criteria in patients with FH is shown in Supplementary Table 1. Patients diagnosed with FH were identified only in the XR+/US + and XR-/US+ groups, whereas no patients were diagnosed with FH in the US- groups. Genetic testing was performed in three patients, all yielding positive results, which were concordant with the clinical diagnoses. Statins were prescribed in 62.8% of patients, ezetimibe in 21.8%, and protein convertase subtilisin-kexin type 9 inhibitor in 1.9%.

**Table 1:** Patient characteristics.

	Overall (*n* = 262)	Concordance (*n* = 218)	Discordance (*n* = 44)	*P* value
Age (years)	75 [65, 81]	74 [64, 81]	77 [71, 81]	0.133
Male, n (%)	221 (84.4)	188 (86.2)	33 (75.0)	0.070
Body mass index (kg/m^2^)	23.5 [21.7, 25.8]	23.5 [21.8, 26.0]	23.4 [20.8, 25.3]	0.460
Hypertension, n (%)	188 (71.8)	156 (71.6)	32 (72.7)	1.000
Dyslipidemia, n (%)	160 (61.1)	130 (59.6)	30 (68.2)	0.314
Diabetes mellitus, n (%)	112 (42.7)	92 (42.2)	20 (45.5)	0.740
History of smoking, n (%)	173 (66.3)	150 (69.1)	23 (52.3)	0.036
Family history of premature CAD (%)	19 (7.7)	16 (7.8)	3 (6.8)	1.000
Clinically diagnosed FH, n (%)	14 (5.3)	10 (4.6)	4 (9.1)	0.264
Renal failure (eGFR < 60 ml/min/1.73m^2^), n (%)	149 (60.3)	118 (55.9)	31 (73.8)	0.039
Hemodialysis, n (%)	16 (6.1)	11 (5.0)	5 (11.4)	0.157
Previous myocardial infarction, n (%)	52 (21.8)	48 (23.9)	4 (10.8)	0.086
Previous PCI or CABG, n (%)	92 (35.1)	79 (36.2)	13 (29.5)	0.489
Acute coronary syndrome, n (%)	88 (34.0)	70 (32.4)	18 (41.9)	0.290
ST-segment elevation MI, n (%)	61 (23.3)	48 (22.0)	13 (29.5)	0.328
Previous ischemic stroke, n (%)	20 (7.6)	13 (6.0)	7 (15.9)	0.054
Laboratory data				
eGFR (ml/min/1.73m^2^)	57.7 [46.0, 70.5]	58.5 [47.0, 71.6]	55.2 [42.7, 62.5]	0.070
Total cholesterol (mg/dL)	161 [134, 198]	159 [134, 197]	168 [150, 205]	0.197
HDL cholesterol (mg/dL)	49 [42, 58]	49 [41, 59]	49 [44, 57]	0.507
LDL cholesterol (mg/dL)	84 [62, 120]	82 [62, 118]	93 [67, 126]	0.231
Triglycerides (mg/dL)	117 [75, 175]	119 [76, 177]	98 [75, 158]	0.362
Hemoglobin A1c (%)	6.1 [5.8, 6.7]	6.1 [5.7, 6.7]	6.2 [5.9, 6.8]	0.512
Medications				
Statin, n (%)	164 (62.8)	140 (64.2)	24 (55.8)	0.305
Ezetimibe, n (%)	57 (21.8)	53 (24.3)	4 (9.3)	0.028
PCSK9-I, n (%)	5 (1.9)	5 (2.3)	0 (0.0)	0.595
Fibrate, n (%)	17 (6.5)	16 (7.3)	1 (2.3)	0.322
Omega-3 fatty acids, n (%)	8 (3.1)	7 (3.2)	1 (2.3)	1.000
Antidiabetic agents, n (%)	83 (31.8)	17 (39.5)	66 (30.3)	0.282
Insulin, n (%)	19 (7.3)	4 (9.3)	15 (6.9)	0.529
AT structural abnormalities, n (%)	40 (15.2)	27 (12.4)	13 (29.5)	0.010

Values are n (%) or median [interquartile range].

AT, Achilles tendon; CABG, coronary artery bypass graft; CAD, coronary artery disease; eGFR, estimated glomerular filtration rate; FH, familial hypercholesterolemia; HDL, high-density lipoprotein; LDL, low-density lipoprotein; MI, myocardial infarction; PCI, percutaneous coronary intervention; PCSK9-I, protein convertase subtilisin-kexin type 9 inhibitor.

### Comparison of AT thickness between XR and US

**Fig. 1:**
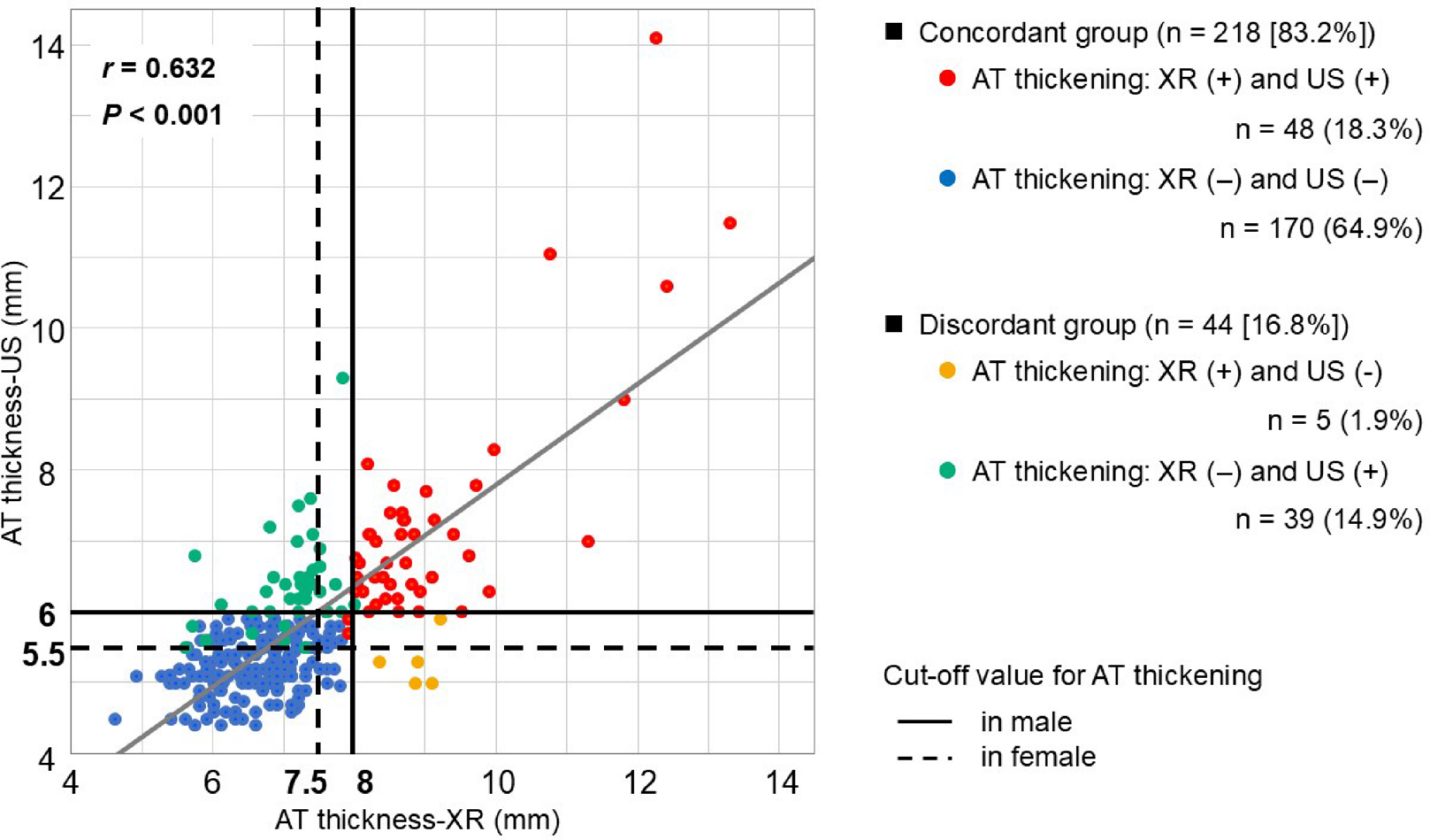
Correlation between Achilles tendon (AT) thickness by soft X-ray radiography (XR) and ultrasonography (US). There was a significant correlation between XR and US for AT thickness measurement (*r* = 0.632, *P* < 0.001). The concordance rate between XR and US for Achilles tendon thickening was 83.2%.

The ICC for interobserver agreement of AT measurements was 0.94 (95% confidence interval [CI] 0.82–0.98) for XR and 0.99 (95% CI 0.96–1.00) for US. The kappa coefficient for interobserver agreement of AT structural abnormalities was 0.93 (95% CI 0.80–1.00). Median differences in AT thickness by XR and US between right and left sides were 0.4 (IQR 0.2–0.7) mm and 0.3 (IQR 0.1–0.5) mm, respectively. In the patient-level analysis, patient representative values across modalities were obtained from the different sides of AT in 39.3% of patients. Median AT thickness by XR was greater than that by US (7.0 [IQR 6.3–7.6] mm vs. 5.8 [IQR 5.1–6.1] mm, *P* < 0.001). The prevalence of AT thickening by XR was lower than that observed by US (20.2% vs. 33.2%) (*P* < 0.001). There was a significant correlation between XR and US for AT thickness measurement (*r* = 0.632, *P* < 0.001). The sensitivity, specificity, positive predictive value, and negative predictive value of US for AT thickening by XR were 90.6%, 81.3%, 55.2%, and 97.1%, respectively. The concordance rate between XR and US for AT thickening was 83.2% (XR + and US+: 18.3% [*n* = 48], XR- and US-: 64.9% [*n* = 170]). Among 44 (16.8%) patients with discordance, XR- and US+ were observed in 14.9% (*n* = 39) and XR + and US- in 1.9% (*n* = 5) (Fig. 1). The agreement between XR and US was moderate (κ = 0.58, 95% CI 0.47–0.69). ROC curve analysis showed AUCs of 0.89 (95% CI, 0.83–0.95) in male and 0.93 (95% CI, 0.86–1.00) in female for US to detect AT thickening defined by XR. The optimal cut-off values for US were 6.0 mm for male and 5.7 mm for female (Supplementary Fig. 1).

**Fig. 2:**
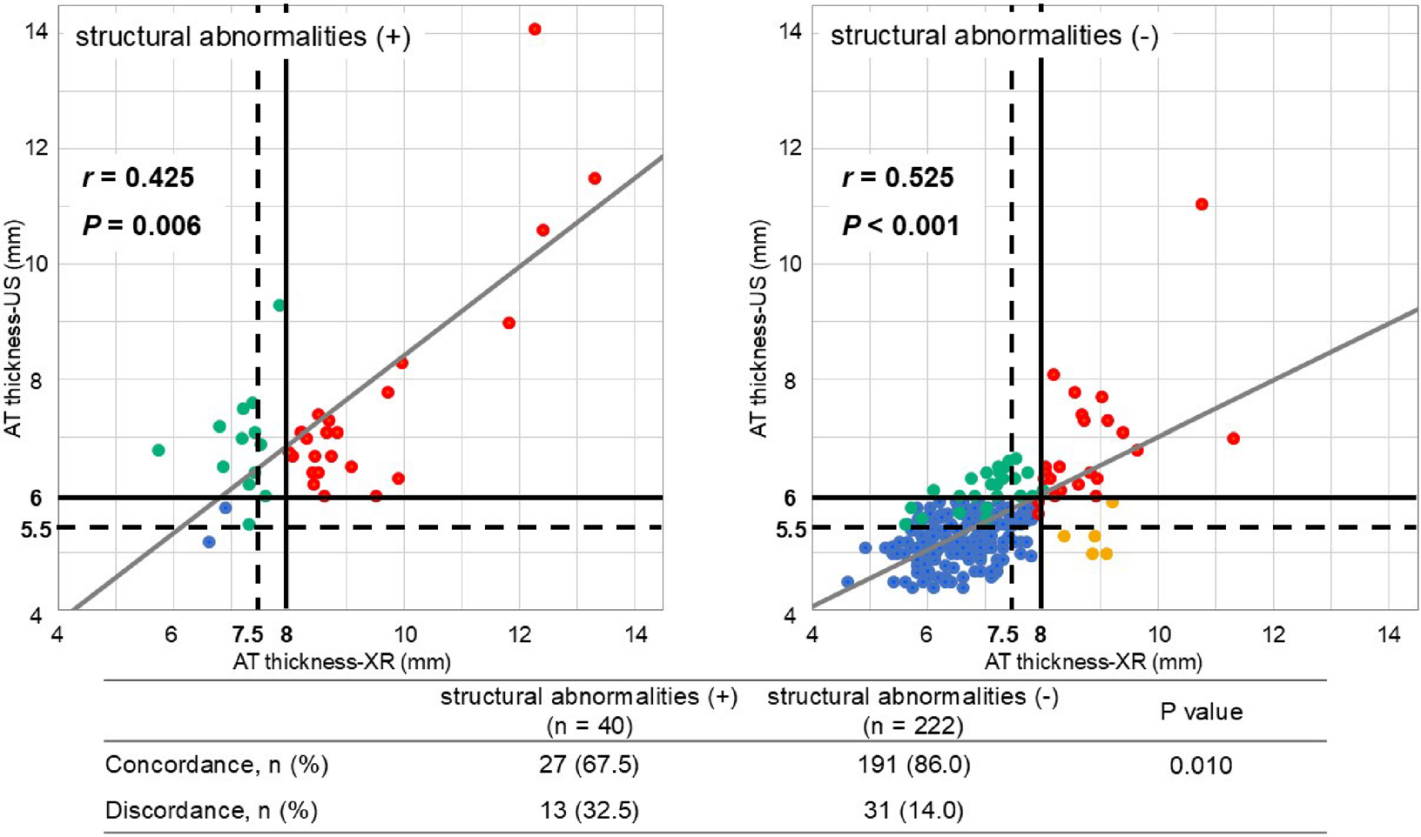
Correlation between Achilles tendon (AT) thickness by soft X-ray radiography (XR) and ultrasonography (US) by the presence of AT structural abnormalities. A significant correlation between XR and US for AT thickness measurement was also observed regardless of the presence of AT structural abnormalities (presence of structural abnormalities: *r* = 0.425, *P* = 0.006; absence of structural abnormalities: *r* = 0.525, *P* < 0.001). The concordance rate significantly differed according to presence of AT structural abnormalities (presence of structural abnormalities: 67.5%, absence of structural abnormalities: 86.0%, *P* = 0.010). AT, Achilles tendon; AT thickness-US, Achilles tendon thickness by ultrasonography; AT thickness-XR, Achilles tendon thickness by soft X-ray radiography; US, ultrasonography; XR, X-ray radiography.

**Fig. 3:**
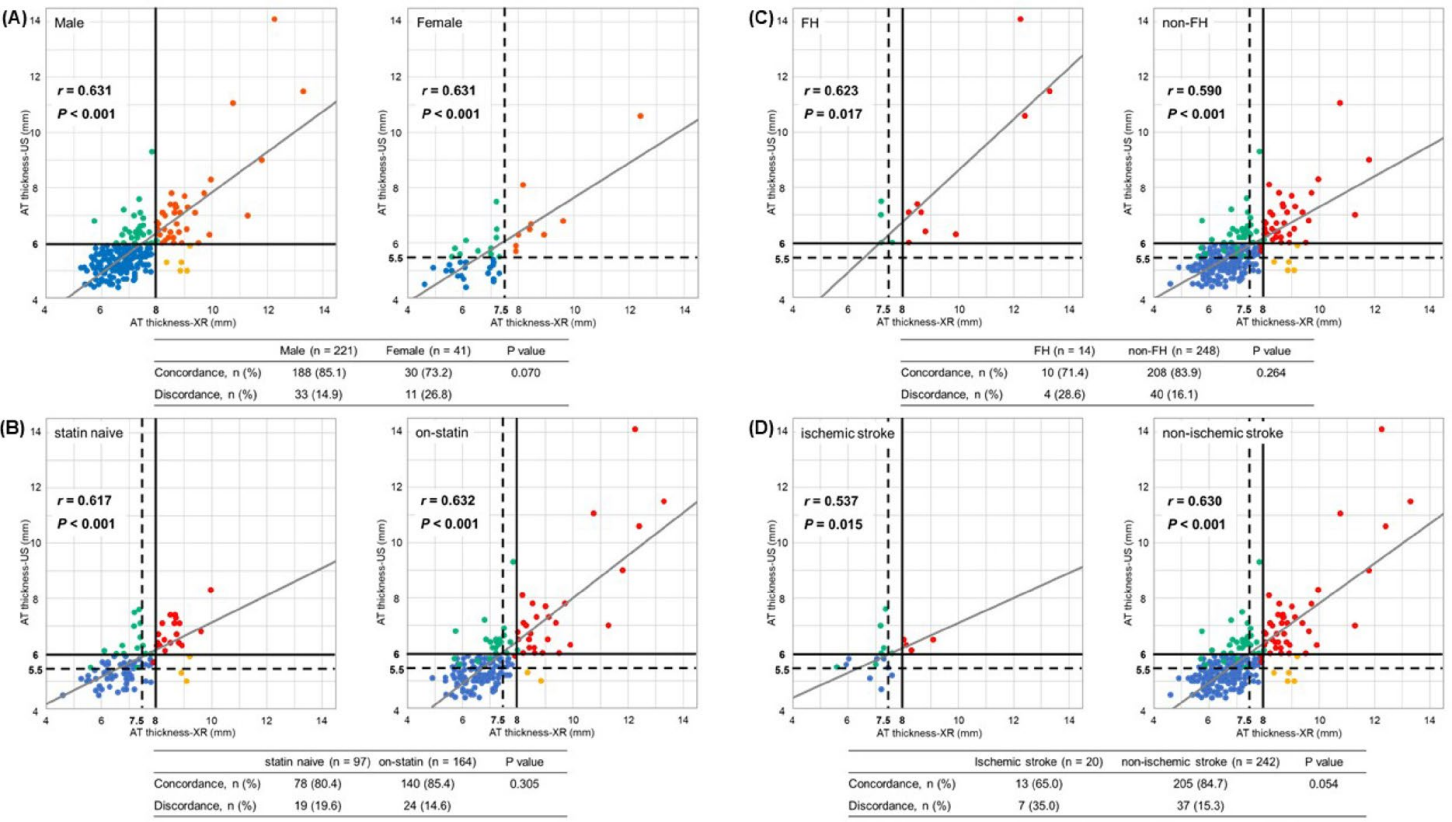
Correlation between Achilles tendon (AT) thickness by soft X-ray radiography (XR) and ultrasonography (US) by sex (**A**), statin use (**B**), the presence of FH (**C**), and previous ischemic stroke (**D**). A significant correlation between XR and US for AT thickness measurement was also observed regardless of sex (male: *r* = 0.631, *P* < 0.001; female: *r* = 0.631, *P* < 0.001), statin use (statin naïve: *r* = 0.617, *P* < 0.001; on-statin: *r* = 0.632, *P* < 0.001), the presence of FH (FH: *r* = 0.623, *P* = 0.017; non-FH: *r* = 0.590, *P* < 0.001), or previous ischemic stroke (ischemic stroke: *r* = 0.537, *P* = 0.015; non-ischemic stroke: *r* = 0.630, *P* < 0.001). The concordance rate between XR and US for AT thickening did not differ significantly between sexes (male: 85.1%, female: 73.2%, *P* = 0.070), statin use (statin naïve: 80.4%, on-statin: 85.4%, *P* = 0.305), the presence of FH (FH: 71.4%, non-FH: 83.9%, *P* = 0.264), and previous ischemic stroke (ischemic stroke: 65.0%, non-ischemic stroke: 84.7%, *P* = 0.054) (**A** and **D**). AT, Achilles tendon; AT thickness-US, Achilles tendon thickness by ultrasonography; AT thickness-XR, Achilles tendon thickness by soft X-ray radiography; FH, familial hypercholesterolemia; US, ultrasonography; XR, X-ray radiography.

**Fig. 4:**
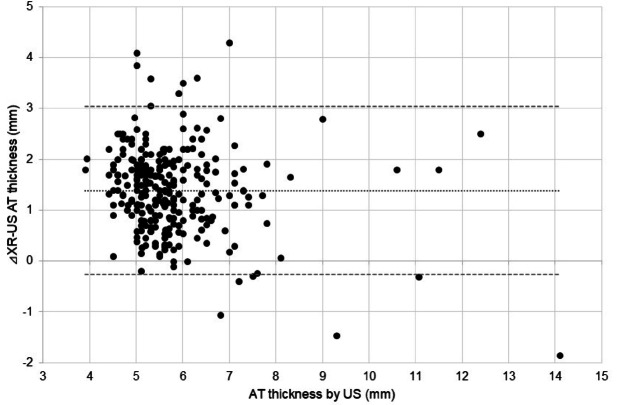
Agreement between soft X-ray radiography (XR) and ultrasonography (US) measurements of Achilles tendon (AT) thickness using the Bland–Altman analysis. The Bland-Altman analysis showed a mean difference of 1.39 mm (XR-US; 95% limits of agreement: − 0.27 to 3.05 mm). AT, Achilles tendon; US, ultrasonography; XR, X-ray radiography.

A significant correlation between XR and US for AT thickness measurement was consistently observed across various subgroups: presence of AT structural abnormalities (presence of structural abnormalities [*n* = 40]: *r* = 0.425, *P* = 0.006; absence of structural abnormalities [*n* = 222]: *r* = 0.525, *P* < 0.001) (Fig. 2), sex (male [*n* = 221]: *r* = 0.631, *P* < 0.001; female [*n* = 41]: *r* = 0.631, *P* < 0.001), statin use (statin naïve [*n* = 97]: *r* = 0.617, *P* < 0.001; on-statin [*n* = 164]: *r* = 0.632, *P* < 0.001), presence of FH (FH [*n* = 14]: *r* = 0.623, *P* = 0.017; non-FH [*n* = 248]: *r* = 0.590, *P* < 0.001), and history of ischemic stroke (ischemic stroke [*n* = 20]: *r* = 0.537, *P* = 0.015; non-ischemic stroke [*n* = 242]: *r* = 0.630, *P* < 0.001) (Fig. 3). The concordance rate between XR and US for AT thickening significantly differed in the subgroup according to presence of AT structural abnormalities (presence of structural abnormalities: 67.5%, absence of structural abnormalities: 86.0%, *P* = 0.010), while the concordance rate did not differ significantly by sex (male: 85.1%, female: 73.2%, *P* = 0.070), statin use (statin naïve: 80.4%, on-statin: 85.4%, *P* = 0.305), FH status (FH: 71.4%, non-FH: 83.9%, *P* = 0.264), or history of ischemic stroke (ischemic stroke: 65.0%, non- ischemic stroke: 84.7%, *P* = 0.054). The Bland-Altman analysis showed a mean difference of 1.39 mm (XR-US; 95% limits of agreement: − 0.27 to 3.05 mm), with XR measurements tending to higher. Values exceeding the upper limit of agreement were observed in AT thickness by US between 5 and 7 mm, a range close to the diagnostic thresholds for AT thickening (i.e. 6 mm in male, 5.5 mm in female) (Fig. 4). In the AT-level analysis, correlation and concordance rate between XR and US were consistent with the results of patient-level analysis except for the subgroup stratified by the presence of ischemic stroke. (Supplementary Figs. 2–5).

### Determinants for discordance between XR and US

Baseline characteristics according to the concordance between XR and US are summarized in Table 1. The prevalence of smoking history, renal failure, and presence of AT structural abnormalities was significantly higher in the discordance group compared with the concordance group. In addition, baseline characteristics across the four groups defined by presence of AT thickening by XR and US are presented in Supplementary Table 2, with significant differences in the prevalence of smoking history, hemodialysis, and AT structural abnormalities.

Multivariable logistic regression analysis demonstrated that presence of AT structural abnormalities was significantly associated with the diagnostic discordance after adjusting for age, sex, and history of smoking (model 1: odds ratio [*OR*] 3.75, 95% CI 1.66–8.47, *P* = 0.001), and age, sex, and renal failure (model 2: *OR* 3.44, 95% CI 1.52–7.81, *P* = 0.003), and age, sex, and previous ischemic stroke (model 3: odds ratio [*OR*] 3.54, 95% CI 1.56–8.05, *P* = 0.003) (Table 2).

**Table 2:** Logistic regression analysis for diagnostic discordance in AT thickening.

Variable	OR (95% CI)	*P* value	Model 1 (*n* = 260)		Model 2 (*n* = 252)		Model 3 (*n* = 261)	
			Adjusted OR (95% CI)	*P* value	Adjusted OR (95% CI) (Model 2)	*P* value	Adjusted OR (95% CI) (Model 3)	*P* value
Age (per year)	1.03 (0.99–1.06)	0.106	1.03 (1.00-1.07)	0.069	1.02 (0.98–1.06)	0.372	1.03 (1.00-1.07)	0.088
Male (Yes vs. No)	0.48 (0.22–1.05)	0.065	0.69 (0.27–1.77)	0.44	0.54 (0.22–1.31)	0.171	0.46 (0.20–1.06)	0.067
BMI (per 1 kg/m^2^)	0.96 (0.87–1.05)	0.358						
Dyslipidemia (Yes vs. No)	1.45 (0.73–2.89)	0.291						
Diabetes mellitus (Yes vs. No)	1.14 (0.60–2.19)	0.691						
History of smoking (Yes vs. No)	0.49 (0.25–0.95)	0.033	0.59 (0.27–1.30)	0.188				
Renal failure (Yes vs. No)	2.22 (1.06–4.65)	0.034			1.82 (0.81–4.06)	0.147		
Previous MI (Yes vs. No)	0.39 (0.13–1.15)	0.086						
Previous ischemic stroke (Yes vs. No)	2.98 (1.12–7.98)	0.029					2.68 (0.95–7.52)	0.062
LDL cholesterol (per 1 mg/dL)	1.00 (1.00-1.01)	0.365						
Use of statin (Yes vs. No)	0.70 (0.36–1.37)	0.299						
AT structural abnormalities (Yes vs. No)	2.97 (1.38–6.36)	0.005	3.75 (1.66–8.47)	0.001	3.44 (1.52–7.81)	0.003	3.54 (1.56–8.05)	0.003

Of the study patients, 99.2% (260/262), 99.2% (252/262), and 99.6% (261/262) were entered into the multivariate models 1, 2, and 3, respectively.

AT, Achilles tendon; BMI, body mass index; CI, confidence interval; FH, familial hypercholesterolemia; LDL, low-density lipoprotein; MI, myocardial infarction; OR, odds ratio.

## Discussion

This is the first study to externally validate the ultrasonographic threshold for AT thickening defined by the JAS FH guideline using XR comparison in patients with CAD. The major findings of the current study were (1) a significant correlation was observed between XR and US in the assessment of AT thickness, with XR consistently resulting in greater values than US, (2) a diagnostic discordance in AT thickening between XR and US was observed in 17% of patients, with the majority showing US positive but XR negative, and (3) AT structural abnormalities were significantly associated with a diagnostic discordance.

In the current study, although the AT thickness measured by XR and US were significantly correlated, the value measured by US is smaller than that by XR with a median difference of 1.2 mm. Previous studies have also consistently shown the greater AT thickness assessed by XR compared with US with the average difference of 1.7–2.1 mm^10–12^. This difference can be explained by several mechanisms. First, the limited ability of XR for the detection of AT border compared with US may lead to the overestimation by XR. Second, the torsion of the AT, which extends from the soleus and gastrocnemius muscles to the calcaneus^13^, and its diagonal alignment relative to the XR beam direction, may be attributable to this difference. Indeed, Sakaguchi et al. have recently reported that, with increasing torsion of AT, a greater discrepancy in AT thickness between XR and US was observed^14^. Of note, the smaller difference in AT thickness between XR and US in the current study (i.e. 1.2 mm vs. 1.7–2.1 mm in previous studies) may be attributable to the predominance of non-FH patients and the relatively lower correlation coefficients in the current study.

Michikura et al. reported in 2017 that the US cut-off values for FH were 5.8 mm (sensitivity 71%, specificity 78%) for male, and 5.5 mm (sensitivity 80%, specificity 81%) for female using data from 130 genetically diagnosed FH patients and 155 non-FH patients^7^. Tada et al. reported in 2021 that the XR cut-off values were 7.6 mm for male (sensitivity 83%, specificity 83%) and 7.0 mm for female (sensitivity 86%, specificity 85%) using data from 987 patients undergoing genetic analyses for FH-genes (including 485 genetically diagnosed FH)^15^. Based on these data, the 2022 JAS FH guideline has revised the XR cut-offs from ≥ 9 mm to ≥ 8 mm in male and ≥ 7.5 mm in female, and newly added the US cut-off values (≥ 6 mm in male and ≥ 5.5 mm in female)^6^. In the current study, the US cut-off values for XR-defined AT thickening based on ROC analysis (6.0 mm in male and 5.7 mm in female) were consistent with those defined by the JAS guideline.

The current study firstly demonstrated that the diagnostic discordance between XR and US was observed in 17% of patients, with the majority showing XR-/US+. In addition to the aforementioned potential explanations for the difference in AT thickness between XR and US, internal structure of AT which can only be assessed by US may also contribute to the observed discordance, particularly in cases with XR-/US+. Indeed, in the current study, multivariable analysis demonstrated a significant association between AT structural abnormalities and diagnostic discordance. Low echoic lesions suggestive of local lipid deposition^16^ are included in the measurement of AT thickness by US; however, XR may not clearly detect lipid components due to their high radiolucency, resulting in a smaller difference in AT thickness measurements between XR and US. This smaller difference may lead to XR-negative but US-positive findings. Although pathogenic variants are identified in only 60–80% of clinically diagnosed FH cases^17^, reflecting the limited sensitivity of genetic testing, further validation of XR and US cutoff values against genetically confirmed FH is required.

From the clinical perspective, clinicians should be aware that the use of US may lead to a higher number of patients diagnosed with FH because US identifies AT thickening more frequently than XR. Although unnecessary genetic testing, increased medical burden, and patient psychological distress related to FH diagnosis and treatment intensification should be carefully considered, the higher sensitivity of US appears acceptable, as the clinical benefits by early initiation and intensification of LDL-C lowering therapy may outweigh these disadvantages. Further research is required to investigate the frequency and clinical relevance of discordance between XR and US for AT thickening.

### Study limitation

There were several limitations in the current study. First, sample size was relatively small, and patients with CAD were only included in the current study, which may limit the generalizability of the study findings. Second, the prevalence of FH may be underestimated because untreated LDL-C levels were not available in around 60% of patients due to baseline statin treatment. Furthermore, because genetic testing was not performed in all patients, a robust reference standard for FH diagnosis was lacking. However, the diagnosis of FH is unlikely to significantly affect the study results. The current study examined the diagnostic discordance between XR and US for AT thickening, rather than determining which modality is superior for diagnosing FH. Further studies should investigate the diagnostic discordance between the modalities in FH patients with genetic diagnosis. Third, representative values across modalities were obtained from different sides of AT in 39.3% of patients, which may be associated with the discordance; however, AT-level analysis showed consistent results. Fourth, although inter-rater reliability was confirmed, the involvement of multiple operators across different institutions in both examination and measurement may have introduced measurement bias. In addition, two different US system were used, and inter-system agreement was not assessed. Finally, given the cross-sectional design of this study, we were unable to assess the downstream clinical implications of potential US overdiagnosis or to develop a clinical decision pathway for managing XR/US discordance. Further investigations are needed to determine the clinical consequences of US overdiagnosis and to formulate practical, evidence-based management strategies for discordant findings.

## Conclusions

Although there was a significant correlation between XR and US for AT thickness measurement, discordance in the diagnosis of AT thickening was observed in 17% of patients. Physicians should be aware that US assessment may result in more patients diagnosed with AT thickening than XR. The cut-off values should be tested in the larger population with a reference standard of FH with genetic diagnosis.

## Supplementary Information

Below is the link to the electronic supplementary material.

Supplementary Material 1

## Data Availability

The datasets generated and/or analyzed during the current study are available from the corresponding author on reasonable request.
