# Supplementary material for: Discordance in diagnostic assessment of Achilles tendon thickening between soft X-ray radiography and ultrasonography among patients with coronary artery disease
Source: Sci Rep. 2026 Apr 21;16:18418. doi: 10.1038/s41598-026-49444-9 (PMC13265914; doi:10.1038/s41598-026-49444-9)
Supplement: Supplementary file 1 — Supplementary Material 1 [file 41598_2026_49444_MOESM1_ESM.pdf]

Supplementary Materials

Discordance in diagnostic assessment of Achilles tendon thickening between soft X-ray radiography and ultrasonography among patients with coronary artery disease

Tadashi Itagaki (MD)[a], Yasushi Ueki (MD, PhD)[a*], Yushi Oyama (MD)[b], Junko Iguchi[a], Koki Fujimori (MD)[a], Daisuke Sunohara (MD)[a], Yuki Yamamoto (MD)[a], Yoshiteru Okina (MD)[a], Hidetomo Nomi (MD, PhD)[a], Tamon Kato (MD, PhD)[a], Tatsuya Saigusa (MD, PhD)[a], Kyuhachi Otagiri (MD, PhD)[b], Soichiro Ebisawa (MD, PhD)[a], Koichiro Kuwahara (MD, PhD)[a].

[a] Department of Cardiovascular Medicine, Shinshu University School of Medicine, Nagano, Japan
[b] Department of Cardiology, Ina Central Hospital, Nagano, Japan

***Author for correspondence:**

Yasushi Ueki, MD, PhD
Department of Cardiovascular Medicine,
Shinshu University Hospital,
3-1-1 Asahi, Matsumoto, Nagano, Japan
e-mail: yasushi522@shinshu-u.ac.jp
Tel. +81 263 37 3352
Fax +81 263 37 2573

<u>Supplementary Figure 1.</u> Receiver operating characteristic (ROC) curve of ultrasonography (US) for Achilles tendon (AT) thickening defined by soft X-ray radiography (XR).

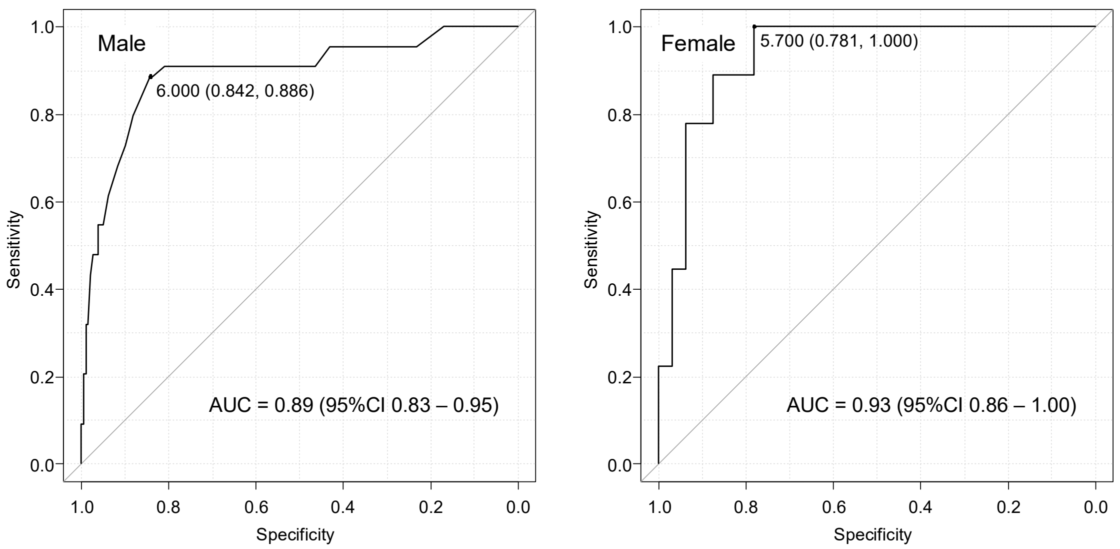

ROC curve analysis showed AUCs of 0.89 (95% CI, 0.83-0.95) in male and 0.93 (95% CI, 0.86-1.00) in female for US to detect AT chickening defined by XR. The optimal cut-off values for US were 6.0 mm for male and 5.7 mm for female.

AT, Achilles tendon; AUC, the areas under the curve; CI, confidence interval; ROC, receiver operating characteristic; US, ultrasonography; XR, X-ray radiography.

<u>Supplementary Figure 2.</u> Correlation between Achilles tendon (AT) thickness by soft X-ray radiography (XR) and ultrasonography (US) at the AT-level.

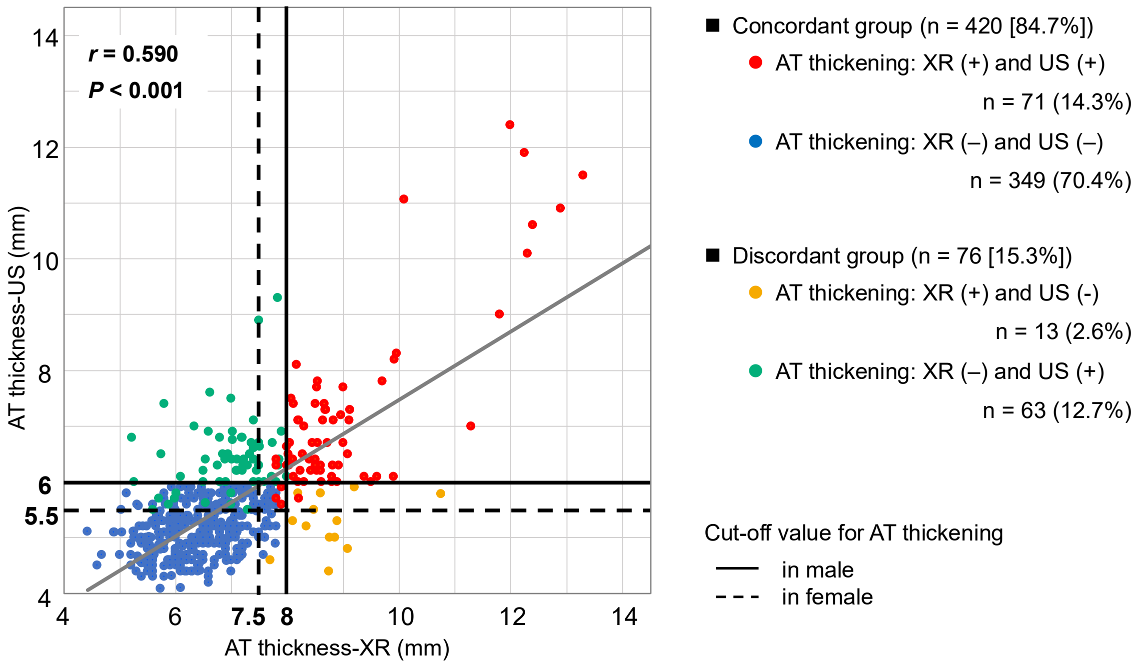

There was a significant correlation between XR and US for AT thickness measurement (r=0.590, P <0.001). The concordance rate between XR and US for Achilles tendon thickening was 84.7%.

AT, Achilles tendon; AT thickness-US, Achilles tendon thickness by ultrasonography; AT thickness-XR, Achilles tendon thickness by soft X-ray radiography; US, ultrasonography; XR, X-ray radiography.

<u>Supplementary Figure 3.</u> Correlation between Achilles tendon (AT) thickness by soft X-ray radiography (XR) and ultrasonography (US) by the presence of AT structural abnormalities at the AT level.

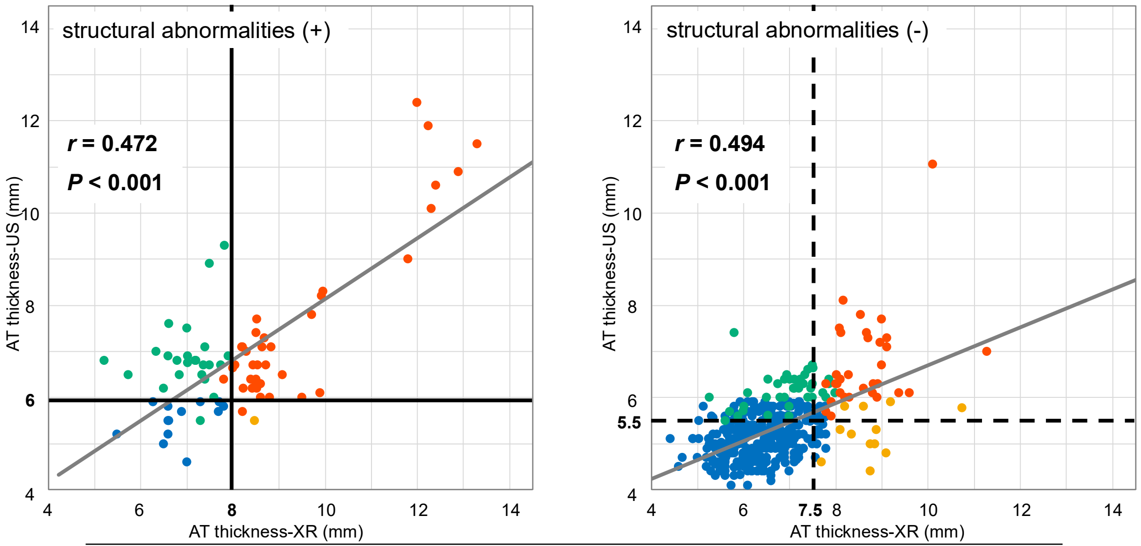

	structural abnormalities (+) (n = 78)	structural abnormalities (-) (n = 420)	P value
Concordance, n (%)	51 (67.1)	369 (78.9)	0.010
Discordance, n (%)	25 (32.9)	51 (12.1)	

A significant correlation between XR and US for AT thickness measurement was also observed regardless of the presence of AT structural abnormalities (presence of structural abnormalities: *r*=0.472, *P* <0.001; absence of structural abnormalities: *r*=0.494, *P* <0.001). The concordance rate significantly differed according to presence of AT structural abnormalities (presence of structural abnormalities: 67.1%, absence of structural abnormalities: 87.9%, *P* <0.010)

AT, Achilles tendon; AT thickness-US, Achilles tendon thickness by ultrasonography; AT thickness-XR, Achilles tendon thickness by soft X-ray radiography; US, ultrasonography; XR, X-ray radiography.

<u>Supplementary Figure 4</u>. Correlation between Achilles tendon (AT) thickness by soft X-ray radiography (XR) and ultrasonography (US) by sex (A), statin use (B), the presence of FH (C), and previous ischemic stroke (D) at the AT level.

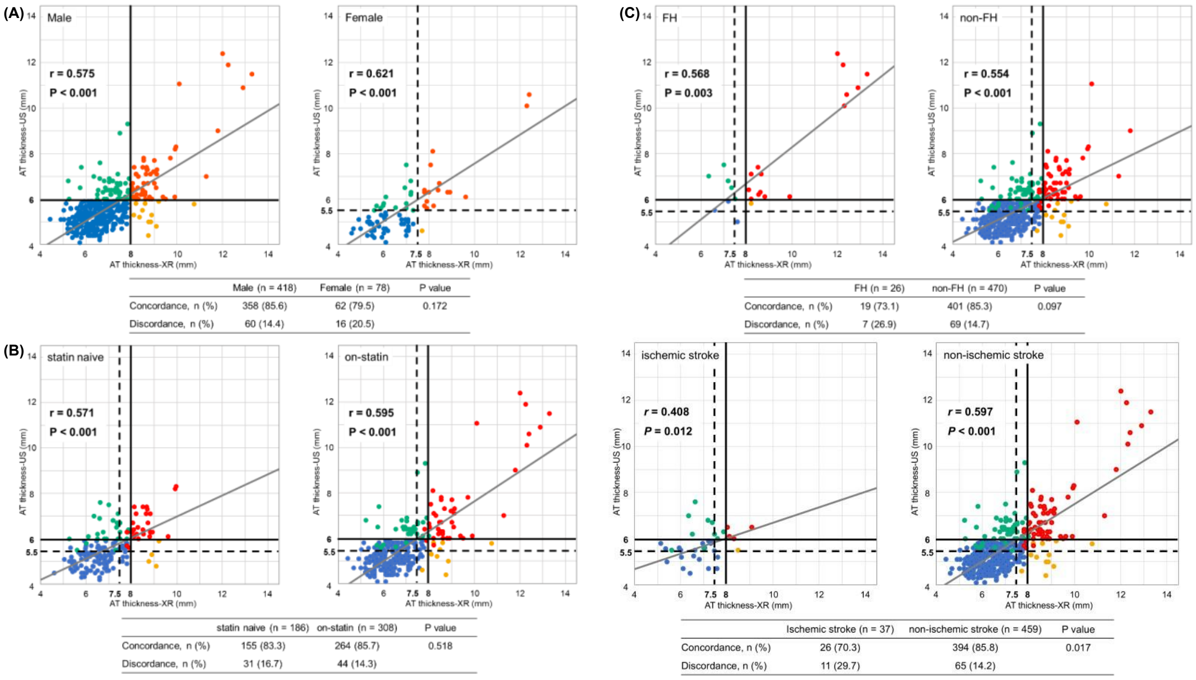

	Male (n = 418)	Female (n = 78)	P value
Concordance, n (%)	358 (85.6)	62 (79.5)	0.172
Discordance, n (%)	60 (14.4)	16 (20.5)	

	FH (n = 26)	non-FH (n = 470)	P value
Concordance, n (%)	19 (73.1)	401 (85.3)	0.097
Discordance, n (%)	7 (26.9)	69 (14.7)	

	statin naive (n = 186)	on-statin (n = 308)	P value
Concordance, n (%)	155 (83.3)	264 (85.7)	0.518
Discordance, n (%)	31 (16.7)	44 (14.3)	

	Ischemic stroke (n = 37)	non-ischemic stroke (n = 459)	P value
Concordance, n (%)	26 (70.3)	394 (85.8)	0.017
Discordance, n (%)	11 (29.7)	65 (14.2)	

A significant correlation between XR and US for AT thickness measurement was also observed regardless of sex (male: r=0.575, P <0.001; female: r=0.621, P <0.001), statin use (statin naïve: r=0.571, P <0.001; on-statin: r=0.595, P <0.001), the presence of FH (FH: r=0.568, P=0.003; non-FH: r=0.554, P <0.001), or previous ischemic stroke (ischemic stroke: r=0.408, P=0.012; non-ischemic stroke: r=0.597, P <0.001). The concordance rate between XR and US for AT thickening did not differ significantly between sexes (male: 85.6%, female: 79.5%, P=0.172), statin use (statin naïve: 83.3%, on-statin: 85.7%, P=0.518), and the presence of FH (FH: 73.1%, non-FH: 85.3%, P=0.097) while a significant difference was observed for previous ischemic stroke (ischemic stroke: 70.3%, non-ischemic stroke: 85.8%, P=0.017).

AT, Achilles tendon; AT thickness-US, Achilles tendon thickness by ultrasonography; AT thickness-XR, Achilles tendon thickness by soft X-ray radiography; FH, familial hypercholesterolemia; US, ultrasonography; XR, X-ray radiography.

<u>Supplementary Figure 5.</u> Agreement between soft X-ray radiography (XR) and ultrasonography (US) measurements of Achilles tendon (AT) thickness using the Bland–Altman analysis at the AT level.

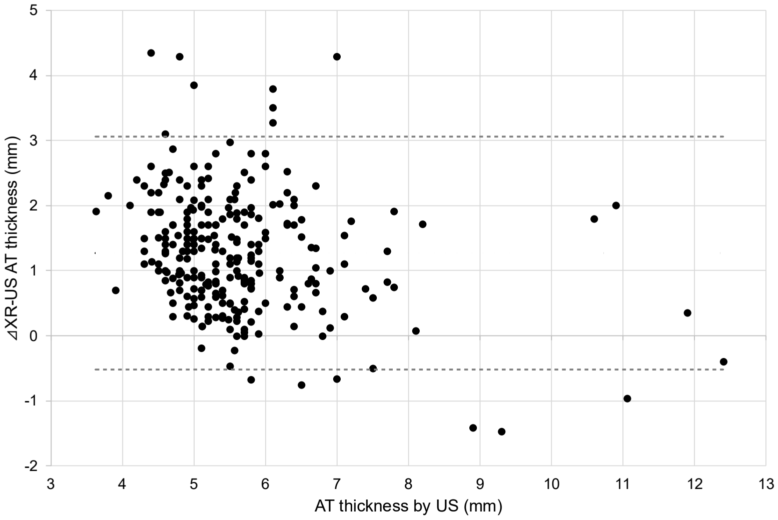

The Bland-Altman analysis showed a mean difference of 1.27 mm (XR-US; 95% limits of agreement: -0.53 to 3.06 mm).

AT, Achilles tendon; US, ultrasonography; XR, X-ray radiography.

Supplementary Table 1. The distribution of diagnostic criteria for FH.

	XR+/US+ (n = 10)	XR+/US- (n = 0)	XR-/US+ (n = 4)	XR-/US- (n = 0)
Elevated LDL-C levels, n (%)	8 (80.0)	NA	3 (75.0)	NA
AT thickening, n (%)	10 (100.0)	NA	4 (100.0)	NA
Family history of FH or premature CAD, n (%)	5 (50.0)	NA	3 (75.0)	NA
Genetic testing for FH, n (%)	3 (30.0)	NA	0 (0.0)	NA

Values are n (%).

AT, Achilles tendon; CAD, coronary artery disease; FH, familial hypercholesterolemia; LDL, low-density lipoprotein; US, ultrasonography; XR, X-ray radiography.

Supplementary Table 2. Patient characteristics across the four groups defined by presence of AT thickening by XR and US.

	XR+/US+ (n = 48)	XR-/US- (n = 170)	XR+/US- (n = 5)	XR-/US (n = 39)	P value
Age (years)	71 [61, 78]	75 [65, 81]	73 [67, 77]	77 [71, 81]	0.080
Male, n (%)	39 (81.2)	149 (87.6)	5 (100.0)	28 (71.8)	0.077
Body mass index (kg/m^2)	23.8 [21.6, 27.3]	23.5 [21.8, 25.6]	25.0 [21.6, 25.6]	23.4 [20.8, 25.3]	0.877
Hypertension, n (%)	31 (64.6)	125 (73.5)	3 (60.0)	29 (74.4)	0.541
Dyslipidemia, n (%)	30 (62.5)	100 (58.8)	3 (60.0)	27 (69.2)	0.682
Diabetes mellitus, n (%)	21 (43.8)	71 (41.8)	3 (60.0)	17 (43.6)	0.889
History of smoking, n (%)	33 (68.8)	117 (69.2)	5 (100.0)	18 (46.2)	0.018
Family history of premature CAD (%)	6 (12.5)	11 (7.1)	0 (0.0)	2 (5.1)	0.593
Clinically diagnosed FH, n (%)	11 (22.9)	0 (0.0)	0 (0.0)	6 (15.4)	<0.001
Renal failure (eGFR < 60 ml/min/1.73m^2), n (%)	29 (61.7)	89 (54.3)	5 (100.0)	26 (70.3)	0.073
Hemodialysis, n (%)	5 (10.4)	6 (3.5)	1 (20.0)	4 (10.3)	0.044
Previous myocardial infarction, n (%)	12 (26.7)	36 (23.1)	1 (20.0)	3 (9.4)	0.253
Previous PCI or CABG, n (%)	17 (35.4)	62 (36.5)	1 (20.0)	12 (30.8)	0.863
Acute coronary syndrome, n (%)	13 (27.7)	57 (33.7)	2 (40.0)	16 (42.1)	0.556
ST-segment elevation MI, n (%)	8 (16.7)	40 (23.5)	2 (40.0)	11 (28.2)	0.404
Previous ischemic stroke, n (%)	4 (8.3)	9 (5.3)	0 (0.0)	7 (18.0)	0.074
**Laboratory data**					
eGFR (ml/min/1.73m^2)	54.7 [43.3, 68.8]	59.0 [50.1, 71.9]	47.0 [40.8, 49.3]	55.9 [44.8, 67.5]	0.050
Total cholesterol (mg/dL)	158 [134, 191]	159 [134, 197]	163 [151, 190]	170 [150, 207]	0.611
HDL cholesterol (mg/dL)	47 [42, 55]	49 [41, 62]	45 [43, 46]	50 [44, 57]	0.602
LDL cholesterol (mg/dL)	88 [68, 121]	81 [61, 116]	91 [42, 119]	95 [75, 126]	0.407
Triglycerides (mg/dL)	122 [69, 187]	117 [78, 177]	94 [93, 208]	99 [74, 155]	0.786
Hemoglobin A1c (%)	6.1 [5.7, 7.5]	6.1 [5.8, 6.6]	6.3 [6.0, 6.6]	6.2 [5.9, 6.8]	0.879
**Medications**					
Statin, n (%)	29 (60.4)	111 (65.3)	2 (40.0)	22 (57.9)	0.536
Ezetimibe, n (%)	11 (22.9)	42 (24.7)	1 (20.0)	3 (7.9)	0.111
PCSK9-I, n (%)	2 (4.2)	3 (1.8)	0 (0.0)	0 (0.0)	0.437
Fibrate, n (%)	3 (6.2)	13 (7.6)	0 (0.0)	1 (2.6)	0.750
Omega-3 fatty acids, n (%)	3 (6.2)	4 (2.4)	0 (0.0)	1 (2.6)	0.450
Antidiabetic agents, n (%)	15 (31.2)	51 (30.0)	2 (40.0)	15 (39.5)	0.664
Insulin, n (%)	6 (12.5)	9 (5.3)	1 (20.0)	3 (7.9)	0.147

AT structural abnormalities, n (%)	25 (52.1)	2 (1.2)	0 (0.0)	13 (33.3)	< 0.001

Values are median [interquartile range], or n (%).

AT, Achilles tendon; CABG, coronary artery bypass graft; CAD, coronary artery disease; eGFR, estimated glomerular filtration rate; FH, familial hypercholesterolemia; HDL, high-density lipoprotein; LDL, low-density lipoprotein; MI, myocardial infarction; PCI, percutaneous coronary intervention; PCSK9-I, protein convertase subtilisin-kexin type 9 inhibitor.
